# Blood Biomarkers in Idiopathic Pulmonary Fibrosis

**DOI:** 10.1007/s00408-017-9993-5

**Published:** 2017-03-28

**Authors:** Julien Guiot, Catherine Moermans, Monique Henket, Jean-Louis Corhay, Renaud Louis

**Affiliations:** Pneumology Department, CHU Liège, Domaine universitaire du Sart-Tilman, B35, B4000 Liège, Belgium

**Keywords:** Idiopathic pulmonary fibrosis, Pulmonary fibrosis, Biomarkers, Interstitial lung disease

## Abstract

**Purpose:**

Idiopathic pulmonary fibrosis (IPF) is a progressive and lethal lung disease of unknown origin whose incidence has been increasing over the latest decade partly as a consequence of population ageing. New anti-fibrotic therapy including pirfenidone and nintedanib have now proven efficacy in slowing down the disease. Nevertheless, diagnosis and follow-up of IPF remain challenging.

**Methods:**

This review examines the recent literature on potentially useful blood molecular and cellular biomarkers in IPF. Most of the proposed biomarkers belong to chemokines (IL-8, CCL18), proteases (MMP-1 and MMP-7), and growth factors (IGBPs) families. Circulating T cells and fibrocytes have also gained recent interest in that respect. Up to now, though several interesting candidates are profiling there has not been a single biomarker, which proved to be specific of the disease and predictive of the evolution (decline of pulmonary function test values, risk of acute exacerbation or mortality).

**Conclusion:**

Large scale multicentric studies are eagerly needed to confirm the utility of these biomarkers.

## Introduction

Idiopathic pulmonary fibrosis is a rare lung disease of unknown origin which leads rapidly to death [1]. Epidemiological studies suggest that the incidence of IPF has been increasing steadily over the last two to three decades [2]. Although the aetiology and the pathophysiology of IPF are still incompletely understood, two anti-fibrotic drugs, pirfenidone and nintedanib, have recently proven to be effective in slowing down disease progression and are now approved as treatments [3, 4]. Clinical management of IPF remains difficult due to a lack of accurate indicators of disease progression, and an absence of simple short-term measures of therapeutic response [5]. Although the median survival is about 2–3 years, there is a wide spectrum of disease courses ranging from slow evolving disease to quick deterioration [1].

Biological markers, often referred as biomarkers, are commonly defined as objectively measured elevated indicators of physiological / pathological processes or pharmacological response to therapeutic interventions [6]. Biomarkers are highly needed in IPF as tools for differential diagnostic, predictor of the progression of the disease and treatment response [7]. Specifically in IPF, an early diagnostic is important to reduce as much as possible the disease progression [8, 9]. Ideally, biomarkers should easily be sampled and analysed for a widespread utility. Therefore, we focused on blood molecules or circulating cells for the simplicity of sampling and processing.

## IPF Definition and Diagnostic Criteria

IPF is defined as a chronic progressive fibrosing interstitial pneumonia of unknown origin [1]. The disease is limited to the lung and associated to a typical radiologic (subpleural and basal predominance, reticulations, honey combing with or without bronchiectasis and no atypical images) and histopathologic pattern (marked fibrosis with architectural distortion, subpleural, patchy involvement of lung, fibroblast foci, absence of inconsistent features) [1]. The diagnosis of IPF also requires the exclusion of other forms of interstitial pneumonia including other idiopathic interstitial lung diseases and ILD’s associated to systemic disease, environmental, exposure or medication. In particular, it is important to exclude chronic hypersensitivity pneumonitis as a hidden cause of IPF by measuring specific IgG [10]. The exploration can be continued by performing a bronchoscopy (with bronchoalveolar lavage), a pulmonary biopsy or a cryobiopsy. All cases have to be discussed by a multidisciplinary group composed of a pulmonologist, a specialist in pulmonary rehabilitation, a rheumatologist, a radiologist, a pathologist and a specialist in occupational medicine.

## Biomarkers (Table 1)

Here we have performed a systematic search in pubmed by typing the words “biomarkers” and “IPF” and selecting those measured in blood. Publications dates of selected papers range from 1993 until 2016.

Many biomarkers have been studied in BALF and in serum as potential diagnostic or prognostic tools. Their utility for monitoring the disease has to be assessed [11].

**Table 1:** Summary of main potential serum biomarkers in IPF

	Biomarker	Diagnostic	Prognosis	Protein level in blood	Ref
Alveolar epithelial markers	KL-6	+	+	Serum baseline level > 1000 U/ml is associated with worse prognosis and >1300 U/ml with increased risk of acute exacerbation	[14, 74]
	SP-A and SP-D	+	+	Increase of 49 ng/mL (1 SD) in baseline SP-A level was associated with a 3.3-fold increased risk of mortality n the first year after presentation. SP-A and SP-D are predictors of worse survival in one year mortality regression model	[24, 25]
Fibrogenesis& extracellular remodelling	MMP-1 & MMP-7	+	+	Higher levels associated to disease progression and worse survival (>4.3 ng/ml for MMP-7)	[30]
	LOXL2	−	+	Higher levels associated with increased risk of progression (>700 pg/ml)	[36]
	Periostin	−	+	Increase level of 116.97 μg/ml levels is associated with disease progression	[39]
	ECM-neoepitope	−	+	Changes in levels are associated with disease progression	[34]
Chemokines	CCL18	−	+	Baseline concentration > 150 ng/ml associated with higher mortality	[44]
	IL-8	+	+	Higher levels associated with worse prognosis (>7.2 pg/ml)	[46, 47]
Growth factors & Adhesion molecules	YKL-40	−	+	High levels (>79 ng/ml) associated with a worse prognosis	[51]
	IGFBP-2	+	Not known	Higher level in IPF reduced in patients with specific anti-fibrotic therapy	[20]
	ICAM-1	−	+	High level associated with worse prognosis (>202.5 ng/ml)	[48, 53]
	VEGF	−	+	Higher levels associated with the disease severity and to predict decline in pulmonary function tests (207 pg/ml)	[56]
Others	HSP70	−	+	The presence of anti-HSP70 IgG is associated with an increase morbi-mortality	[58, 75, 76]
	Leptin	−	+	Worse survival when >13.79 ng/ml in case of acute exacerbation	[60]
	CXCL13	+	+	Higher levels is associated with a worse prognosis	[67, 77]
Circulating cells	T-cells (Sema7a /CD28)	+/−	+	Reduce expression of CD28 or increase Sema7a + Treg is associated with a higher mortality	[62, 65, 78]
	Fibrocytes	−	+	Elevated circulating fibrocytes (>5%) is associated with an early mortality	[68]

*KL-6* Krebs von den lungen-6 antigen, *SP-A* Surfactant protein A, *SP-D* Surfactant protein D, *MMP-1* Matrix metalloproteinase-1, *MMP-7* Matrix metalloproteinase-7, *LOXL2* lysyl oxidase-like 2, *CCL18* CC chemokine ligand 18, *IL-8* interleukin-8, *IGFBP-2* insulin-like growth factor binding protein-2, *HSP70* Heat shock protein 70, *CXCL-13* C-X-C motif chemokine 13

### Alveolar Epithelial Markers

The main biomarkers associated with the alveolar epithelial cell damage (or dysfunction) are the Krebs von den Lungen-6 (KL-6) antigen and the surfactant protein A and D (SP-A and SP-D). Compared to SP-A and SP-D, serum KL-6 has a highest accuracy for diagnosis of interstitial lung diseases (IPF and ILD associated with connective tissue diseases) [11].

#### Krebs von den Lungen-6 Antigen (KL-6)

KL-6 is a high molecular weight glycoprotein classified as a human transmembrane mucin 1 (MUC1). That glycoprotein is mainly expressed at the extracellular membrane surface of type II pneumocytes [12–14]. It has been initially studied in non-IPF interstitial lung diseases (ILDs) and appeared to be also elevated in IPF with a possible correlation with an increased risk of IPF associated mortality [14, 15]. KL-6 promotes migration, proliferation and survival of lung fibroblasts [16, 17] and therefore is possibly involved in the IPF pathophysiological process. A serum baseline level above 1000 U/ml seems to be associated with a worse outcome [18, 19]. Our data indicate that serum KL-6 levels are markedly increased in IPF as compared to healthy subjects but we did not find reduced KL-6 levels in the patients treated by pirfenidone or nintedanib [20]. Serum KL-6 could possibly be associated to treatment response but this needs further longitudinal studies to be confirmed.

#### Surfactant Protein A and D (SP-A and SP-D)

Surfactant proteins are lipoprotein complexes synthesized and secreted by type II pneumocytes to decrease surface tension at the air–liquid interface. They have also a role in the lung host defence. Serum SP-A and SP-D are known as being elevated in patients with ILD with a greater extend in IPF [21–25]. Some findings also suggest that surfactant lipids may protect against intraluminal fibrogenesis by inducing fibroblast apoptosis and decreasing collagen accumulation [26]. In this context, SP-A and SP-D have been shown to be predictive of survival when assessed at the initial work up of the disease [24, 27]. Their involvement in the IPF physiopathological process is underlined by familial form of IPF associated with mutations of surfactant protein C [28].

### Fibrogenesis and Extracellular Remodelling Markers

The main biomarkers identified in IPF focusing on fibrogenesis are matrix metalloproteinases (MMP)-1 and -7, Lysyl oxidase-like 2 (LOXL2) and Periostin.

#### Matrix Metalloproteinases-1 and -7 (MMP-1 and MMP-7)

Matrix metalloproteinases are a collection of zinc-dependent proteases involved in the breakdown and the remodelling of extracellular matrix components [29]. MMP-1 and MMP-7 seem to be primarily overexpressed in plasma of IPF patients compared to hypersensitivity pneumonitis, sarcoidosis and COPD with a possible usefulness in differential diagnosis [30]. They are also involved in inflammation and seem to take part to the pathophysiological process of pulmonary fibrosis [31, 32]. The most studied is clearly MMP-7 which is known as being significantly increased in epithelial cells both at the gene and protein levels and is considered to be active in hyperplastic epithelial cells and alveolar macrophages in IPF [33]. There is also a significant correlation between higher MMP-7 concentrations and disease severity assessed by forced vital capacity (FVC) and DLCO (%pred) [30]. We therefore can identify MMP-7 as one of the greatest individual biomarkers in IPF.

Concentrations of protein fragments generated by MMP activity are increased in the serum of individuals with IPF compared with healthy controls. Increased neoepitope concentrations were associated with disease progression as defined by death or decline in FVC > 10% at 12 months after study enrolment and the rate of this increase predicted survival [5, 34]. We can hypothesize that as neoepitopes are generated by proteases they could be associated with lung remodelling and fibrotic process.

#### Lysyl Oxidase-like 2 and Periostin

Lysyl oxidase-like 2 (LOXL2) is expressed in fibrotic lung and is thought to play a crucial role in matrix remodelling and fibrogenesis [35]. Serum level of LOXL2 is correlated to IPF disease progression (assessed by classical regression tree method) [36]. Nevertheless, anti-LOXL2 targeted therapy trial failed to reduce IPF disease progression [37]. Periostin, an extracellular matrix protein that contributes to fibrosis in the lung, is highly elevated in blood of IPF patients as well as in lung tissue [38]. In lung tissue, it is specifically found in the “fibroblast foci”, typically seen in IPF. Plasma level of periostin is also correlated with a composite score reflecting disease progression [39].

### Chemokines

#### CC Chemokine Ligand 18

CC motif chemokine ligand 18 (CCL18) is a small protein derived from alveolar macrophages that acts as a chemo-attractant. CCL18 is mainly secreted by antigen-presenting cells such as monocytes, macrophages and dendritic cells [40]. In the setting of pulmonary fibrosis, alveolar macrophages are believed to be the main source of CCL18 in the lung and play a role in the pathogenesis of pulmonary fibrosis [41]. Serum CCL18 is increased in IPF but is not specific of the disease [41–43]. In IPF, CCL18 is negatively correlated to pulmonary function tests (TLC and DLCO) [42]. In a prospective study, it has been shown that patients with serum level of CCL18 > 150 ng/ml were independently associated with death in IPF (HR 1.98, 95% CI 2.49–25.51, *p* = 0.005) [44]. Moreover, pirfenidone one of the specific anti-fibrotic therapies in IPF significantly suppressed the expression of CCL18 on macrophages [45]. Therefore, CCL18 could have a potential interest as a prognostic tool in IPF.

#### Interleukin-8 (IL-8)

IL-8 is a cytokine, which is highly chemo-attractant for neutrophils and known to be elevated in serum of IPF patients. A study identified a negative correlation between IL-8 and pulmonary function tests (DLCO, TLC, VC) [46, 47] and survival [48] underlying its potential utility as biomarker in IPF.

### Growth Factors and Adhesion Molecules

#### YKL-40

YKL-40 is a chitinase-like protein that regulates cell proliferation and survival and has previously been described in liver fibrosis. YKL-40 has also been well studied in multiple ILDs [13]. Its mechanism of action is not understood yet but seems to be associated with a promitogenic action on lung fibroblasts in animal model and increases macrophages activity in COPD [49, 50]. Moreover, YKL-40 has been found to be increase in fibrotic areas of IPF patients and especially in macrophages and bronchial cells [13]. In a prospective study, YKL-40 is not specific for IPF but a serum level above 79 ng/ml is associated with a worse prognosis (HR 10.9, 95% CI 1.9–63.8, *p* < 0.01) [51]. Therefore, we think that YKL-40 is useful at diagnosis for prognosis stadification and could be considered.

#### Insulin-like Growth Factor 2 (IGFBP-2)

IGFBP-2 is a member of a highly conserved family of six insulin-like growth factor (IGF) binding proteins, which has recently been identified in IPF [20, 52]. IGF and IGFBPs are described to be involved in cell proliferation and differentiation. In a cross sectional study on 50 patients suffering from IPF, we have recently shown that IGFBP-1 and IGFBP-2 are increased in newly diagnosed IPF and IGFBP-2 reduced in patients treated with anti-fibrotic therapy though still raised as compared to healthy subjects [20]. However, we did not report any correlation between IGFBP-2 and impaired lung function indices. In contrast to IGFPB-1 and -2, it turned out that IGF-1 and -2 were decreased in serum of untreated IPF. Further, longitudinal studies are needed to evaluate their usefulness as biomarkers in IPF.

#### ICAM-1 and ICAM-2

Intracellular adhesion molecule-1 and -2 (ICAM-1 and ICAM-2) are also elevated (not specifically) in serum from patient with IPF. ICAM-1 has been found to be overexpressed on pulmonary epithelial cells of patients with IPF [53, 54], whereas ICAM-2 has been inversely associated with DLCO. Its utility in clinical practice is not yet known and has to be explored in longitudinal studies.

#### VEGF

Vascular endothelial growth factor (VEGF) is a glycoprotein expressed in alveolar epithelial cells [55]. Increased serum VEGF, but not CRP, has been identified in one longitudinal study of 41 IPF patients and potentially associated with the disease severity as reflected by the alveolo-arterial difference of O_2_. In this study, serum levels of VEGF in severely hypoxemic IPF were similar to those found in non-small cell lung carcinoma [56]. Serum VEGF was associated with increased loss in VC over time. Survival rate at 5 years was decreased in patients whose VEGF level was above 207 pg/ml. Of interest, VEGF is targeted by nintedanib used as a specific IPF therapy [57].

### Others

The heat shock protein 70 (HSP 70) is a molecular chaperone that is expressed in response to stress. IgG autoantibodies directed to HSP70 have been associated with a poorer prognostic in IPF [58]. In addition, Fibulin-1 is elevated in IPF and correlated with the disease progression [59]. Plasma leptin level, a biomarker traditionally associated with obesity, is also significantly increased in case of acute exacerbation of IPF. In one longitudinal study of 61 IPF patients, those patients with plasma leptin levels > 13.79 ng/ml at hospital admission had increased mortality [60].

A recent study focusing on biomarkers that might assist in distinguishing IPF from non-IPF-ILDs have identify in the plasma of 149 IPF patients (derivation cohort *n* = 86, validation cohort *n* = 63) that a biomarker index composed of SP-D, MMP-7 and osteopontin enhanced diagnostic accuracy for IPF compared to other ILDs (rheumatoid arthritis associated ILD (*n* = 33); alternative idiopathic ILD (*n* = 41)) [61]. This three analyte panel of SP-D, MMP-7 and osteopontin enhance the odds of IPF diagnosis when each of them exceed its threshold value (>31 ng/ml, >1.75 ng/ml and >6 ng/ml, respectively) [61].

## Circulating Cells

T-cells are also involved in the pathophysiology of IPF. Semaphorin 7a (Sema 7a), a membrane-bound protein expressed in activated Tcells, also regulates inflammatory responses via stimulation of macrophage chemotaxis and cytokine production chemokine expression, modulation of T-cell function and regulation of collagen production by fibrocytes. Hematopoietic expression of Sema 7a is sufficient to induce fibrosis in TGF-β1–induced murine lung fibrosis [62]. There is an increased expression of Sema7a^+^ on T-regulatory cells in IPF [62–64]. Its protective or deleterious impact in IPF is not known yet. By contrast, CD28 (a marker of T-cell activation) is less expressed in IPF with a higher incidence of mortality in case of low expression [65]. As it has been shown in several auto-immune disease, there is a lower expression of CD4^+^CD25^+^FOXP3^+^ T reg cells in IPF [66]. Other teams have been focusing on B-cell activation using BLys (B-cell activating factor) and CXCL13, which underlined the potential implication of B-cells in IPF [67].

Circulating CD45^+^Col-1^+^ fibrocytes are circulating bone-marrow derived mesenchymal progenitor cells which can differentiate into fibroblast and myofibroblasts [68]. They are increased in IPF compared to healthy subjects. Their circulating level in IPF (>5% of total blood leukocytes) is associated with a worse survival [69, 70].

## Further Approach

Genomic, epigenetic and molecular phenotyping are widely used in clinical research. It is getting more and more clear that genetic polymorphisms, whole blood transcriptomic profile, as well as lung microbiome are predicting groups of diverse disease behaviour and response to treatment [71]. Extracellular vesicles containing micro-RNA are known to express surface protein and hold nucleic acid, including microRNAs, and to regulate gene expression in the recipient cells. A recent study identified miR-21-5-p as to be elevated in the serum of IPF and associated to the disease progression and the overall prognosis [72]. Many other miR have also been identified like miR-7, miR-29 and miR-125b [73].

## Limitations

One of the limitations of our review is that we have chosen candidate biomarkers in IPF according to our thinking and their potential usefulness. Another limitation is the lack of longitudinal studies for some of the biomarkers, which reduces the clinical impact of the finding because of a lack of assessment of treatment response. We believe that further longitudinal multicentre studies are highly needed to evaluate the real clinical impact of most of the biomarkers in a single or multivariate analysis as diagnostic and prognostic tools.

## Conclusion

Diagnosis of IPF remains challenging and requires a multidisciplinary approach. Treatments currently available have a limited efficacy and the overall prognosis of the disease remains poor. Recent advance in pathophysiological process are promising for further therapies; nevertheless, the most important point for now is the early diagnosis as a mean to early treatment. So far, there is no easy to use biomarker available for clinical practice. It is now obviously clear that genetic and epigenetic variations are involved in the pathogenesis giving us different information for the morbi-mortality in IPF. Furthermore, prospective longitudinal studies have started to identify blood biomarkers to be used in clinical practice.
